# Draft genome sequence of *Bacillus* sp. strain X and *Salarachaeum* sp. strain III isolated from Lake Karum, Danakil Depression, Ethiopia

**DOI:** 10.1128/mra.01260-23

**Published:** 2024-02-22

**Authors:** Ermias Sissay Balcha, Karen Olsson-Francis, Ben Stephens, Barbara Cavalazzi, Adugna Abdi Woldesemayat, Mesfin Tafesse Gemeda, Michael C. Macey

**Affiliations:** 1 School of Medical Laboratory Science, College of Health Sciences, Hawassa University, Hawassa, Ethiopia; 2 Department of Biotechnology, College of Biological and Chemical Engineering, Addis Ababa Science and Technology University, Addis Ababa, Ethiopia; 3 AstrobiologyOU, School of Environment, Earth and Ecosystem Sciences, The Open University, Milton Keynes, United Kingdom; 4 Dipartimento di Scienze Biologiche, Geologiche e Ambientali, Università di Bologna, Bologna, Italy; 5 Department of Geology, University of Johannesburg, Johannesburg, South Africa; Indiana University, Bloomington, Bloomington, USA

**Keywords:** genome sequences, extreme environments, Lake Karum

## Abstract

Here, we report the draft genome sequences of strains of *Bacillus* and *Salarachaeum* that were isolated from hypersaline water samples collected from Lake Karum, Danakil Depression, Ethiopia. The sequences pave the way for more targeted studies into the potential biological activities and secondary metabolite synthesis of these organisms.

## ANNOUNCEMENT

Lake Karum, also known as Lake Assale, is located in the Danakil Depression, northern Ethiopia. It is a brine lake (28%–33% salinity) that lies 120 m below sea level with temperatures ranging between 34°C and 36°C (1, 2). Previous studies have shown that Lake Karum supports a diverse microbial community (3, 4). Here, we present the draft genomes of *Bacillus* sp. strain X and *Salarachaeum* sp. strain III. The strains were isolated from water samples collected using falcon tubes from a depth of 5 cm in Lake Karum (14.0207° N, 40.4007° E).

The water samples were enriched with Soya Flour Medium (SFM) containing 4 M NaCl. Serial dilutions were prepared and plated onto soy flour agar and incubated at 30°C for 30 days. Distinct colonies were observed, leading to isolation of *Bacillus* sp. strain X and *Salarachaeum* sp. strain III (5). Pure colonies were used to culture in SFM, from which DNA was extracted using Griffiths technique (6). DNA libraries were prepared by MicrobesNG using Nextera XT Library Prep Kit. Paired-ends (2 × 250 bp) genomic sequencing was carried out by MicrobesNG (Birmingham, UK) using Illumina HiSeq technology. Raw reads were trimmed using Trimmomatic (v0.30) (quality cutoff of Q15) (7), and *de novo* assembly was carried out with SPAdes (v3.7) (8). Genome quality was assessed with CheckM (9). Genome coverage was calculated using BWA, SAMtools (0.1.19), and BEDTools genomecov (2.2.7) (10–12). Genome annotation was performed using the Rapid Annotations Subsystems Technology (RAST) annotation server (v2.0) with the classic RAST pipeline (13). The presence of metabolic pathways was further screened using BlastKoala (2.3) (14).

*Bacillus* sp. strain X was most closely related to *Bacillus subtilis*, with an average nucleotide identity of 98.26% as determined by ANItools (15). The draft genomes of *Bacillus* sp. strain X yielded 17 contigs, including 2,322 coding sequences (CDSs), 44.92% GC content, and 3,391,284 bp genome size, with 160-fold coverage. *Salarachaeum* sp. strain III X was closely related to Halobacteriales, with 91.36% nucleotide sequence identity. The genome comprised 129 contigs, including 943 CDSs with 67.50% GC content. The genome size was 2,034,915 bp with 30-fold coverage. CheckM analysis identified completion and contamination values of 98.73% and 0.32% for the *Bacillus* genome and 99.28% and 1.52% for the *Salarachaeum* genome.

Genome mining of *Bacillus* sp. strain X was carried out with antibiotics and Secondary Metabolites Analysis SHell (antiSMASH, v6.0) program (16) and showed the presence of 13 (44.8 %) non-ribosomal peptide gene clusters that encoded a variety of secondary metabolites that produce enzymes, putative antimicrobials, and other bioactive compounds of significant importance in the biotechnological and medical sectors (17). Gene clusters encoding ribosomally synthesized and post-translationally modified peptides and terpenes were also identified from *Salarachaeum* sp. strain III. Regarding biogeochemical cycling, genes for the complete assimilatory sulfate and dissimilatory nitrate pathways were identified in the genome of *Bacillus* sp. strain X, while the genome of *Salarachaeum* sp. strain III contained putative formate dehydrogenase (NAD-dependent) and carbon monoxide dehydrogenase-encoding genes.

## Data Availability

The Whole Genome Shotgun project has been deposited at DDBJ/ENA/GenBank under the accession no. PRJNA1050408 for Bacillus sp. strain X and JAZHPG000000000 for Salarachaeum sp. strain III. The version described in this paper is the first version.
